# Characterizing Low-Risk Breast and Gynecological Cancer Patients for Transition into an Oncology/Primary Care Coordinated Care Model: Findings from a Survey of Diverse Survivors in a Rural U.S. State

**DOI:** 10.3390/cancers13174428

**Published:** 2021-09-02

**Authors:** Miria Kano, Lu Chen, Tawny Boyce, Ricardo Gomez, Amy C. Gundelach, Shoshana Adler Jaffe, Andrew L. Sussman, Zoneddy R. Dayao, Jolene Lobo, Claire R. Pestak, Teresa L. Rutledge

**Affiliations:** 1 Department of Internal Medicine, University of New Mexico, Albuquerque, NM 87131, USA; ZDayao@salud.unm.edu (Z.R.D.); ClPestak@salud.unm.edu (C.R.P.); 2 The University of New Mexico Comprehensive Cancer Center, Albuquerque, NM 87131, USA; TBoyce@salud.unm.edu (T.B.); ricagomez@salud.unm.edu (R.G.); AGundelach@salud.unm.edu (A.C.G.); SAdlerjaffe@salud.unm.edu (S.A.J.); ALSussman@salud.unm.edu (A.L.S.); TRutledge@salud.unm.edu (T.L.R.); 3 Department of Preventive Medicine and Population Health, The University of Texas Medical Branch; Albuquerque, NM 77555, USA; Luchen1@utmb.edu; 4 Department of Family and Community Medicine, University of New Mexico, Albuquerque, NM 87131, USA; JLobo101@salud.unm.edu; 5 Department of Obstetrics and Gynecology, University of New Mexico, Albuquerque, NM 87131, USA

**Keywords:** survivorship care, rural and underserved, cancer disparities, breast and gynecological cancers, cancer care delivery

## Abstract

**Simple Summary:**

We conducted a survey to identify the key aspects that influence cancer care for racial/ethnic and geographically diverse low-risk breast and gynecologic cancer patients. New Mexico is a large state with many ethnic and racially diverse communities who reside in rural areas. Data obtained through our New Mexico Tumor Registry show that these patients often have different and worse cancer health outcomes. To learn more about how to improve their care, we surveyed patients regarding their access to primary care (PC); compliance with screening recommendations; treatment for conditions other than cancer (e.g., diabetes, obesity, heart problems, etc.); difficulties attending their clinic visits; and whether or not they received information about their survivorship care in the form of a survivorship care document (SCD). We found that the majority of the 150 patients surveyed reported having a Primary Care Provider (PCP). Many had health complications other than cancer, those who resided in rural areas had more difficulties getting to their cancer follow-up appointments, and nearly half had not received SCDs. We used these survey results to develop an oncology/PCP care coordination intervention to improve the oncology and cancer survivorship care for those who were at low risk of recurrence.

**Abstract:**

We conducted a survey to characterize the key attributes of racial/ethnic and geographically diverse low-risk breast and gynecologic cancer patients. We collected data regarding patients’ access to primary care (PC); compliance with screening recommendations; treatment for comorbidities; logistical barriers to clinic visits; and receipt of survivorship care documentation (SCD). Survey findings informed the development of an oncology/Primary Care Provider (PCP) care coordination intervention to improve care. We distributed a cross-sectional survey among a convenience sample of 150 cancer survivors. Responses were calculated using descriptive statistics and compared based on the distance participants traveled to their appointments at the cancer center (≤30 vs. >30 miles). Of the 150 respondents, 35% traveled >30 miles for follow-up care and 78% reported having one or more comorbid condition(s). PC utilization was high: 88% reported having a PCP, and 91% indicated ≤1 yearly follow-up visit. Participants traveling >30 miles reported higher rates of logistical challenges associated with cancer center visits compared to those traveling ≤30 miles. Nearly half of respondents (46%) had not received SCD. In conclusion, survey studies such as these allow for the systematic assessment of survivor behaviors and care utilization patterns to inform the development of care coordination interventions for diverse, low-risk cancer patients.

## 1. Introduction

As the population in the United States (U.S.) grows, diversifies, and lives longer, the number of cancer survivors is expected to increase from 15.5 million (2016) to an estimated 20.3 million by 2026 [1]. Cancer survivors require surveillance for recurrent and subsequent malignancies, management of long-term and late effects of cancer treatment, consistent care to address high rates of comorbid conditions, health promotion, and care coordination [1,2,3,4]. However, such cancer follow-up care is provided inequitably across cancer survivor populations. Studies document cancer health disparities across the cancer care continuum (i.e., from prevention through survivorship) among racial ethnic minority populations, including Native American/Alaskan, [5] Hispanic/Latinx, and African American populations. [6] Racially and ethnically driven cancer disparities are intensified for residents of rural and frontier areas [7]. A recent national cross-sectional survey shows that upon completion of cancer treatment, patients, in rural and underserved areas, experience unmet physical and psychosocial needs across sixteen survivorship domains, as well as suboptimal rates of preventive and cancer surveillance screening.

In response to this looming crisis in cancer care delivery, the American Society of Clinical Oncology (ASCO) drew on a pivotal 2005 Institutes of Medicine (IOM) report [3], proposing a multifaceted strategy to redesign service delivery models by expanding the role of primary care providers (PCPs) in follow-up cancer care [8]. Enhanced integration of PCPs in survivorship care for low-risk cancer patients, many of whom continue to receive routine follow-up care from an oncologist despite being well from a surveillance perspective, would shift a larger proportion of survivorship care from oncology settings into primary care sites. However, no evidence-based models for care coordination exist to assist providers seeking to integrate oncology and primary care for their patients [9,10,11,12,13,14,15]. Furthermore, little is known about patient access to primary care, primary care provider training, and/or access to cancer treatment resources required to make such coordinated care efforts feasible and sustainable [9,16,17,18,19].

The University of New Mexico Comprehensive Cancer Center (UNMCCC), the only National Cancer Institute Designated Cancer Center within a 500-mile radius, serves a diverse multiethnic and multicultural population dispersed across the nation’s fifth largest state in terms of landmass. New Mexico has the highest percentage of Hispanics and Native Americans of any state, as 49.1% of the total population are Hispanic or Latinx, 10.9% American Indian, 2.6% African American, 1.6% Asian, and 37.1% are non-Hispanic or White [20]. Persistent poverty is manifest in per capita income, childhood poverty, food insecurity, and high school graduation rates. The healthcare system suffers from shortages of oncologists and PCPs [21]. Patients in rural areas have limited access to specialized cancer care. Approximately 40% of UNMCCC patients reside outside Bernalillo County (where UNMCCC is located), some traveling up to a 300-mile roundtrip for treatment [22]. To address these patients’ needs and oncology service deficits, and assess the degree to which cancer patients have access to primary care in compliance with cancer follow-up and cancer screening, we conducted a cross-sectional survey of breast and gynecological cancer patients/survivors. In this short communication, we present the results of this cross-sectional survey, considering the ways in which patient self-reported access to, and utilization of, primary care services may facilitate the effective modeling of oncology/PCP care coordination to better serve the ethnically/racially and geographically diverse cancer survivors across this vast state.

## 2. Materials and Methods

### 2.1. Cross-Sectional Survey Data and Sample

We conducted a cross-sectional survey among a convenience sample of breast and gynecologic cancer survivors with low risk of recurrence (Breast: stages 0,1,2; Gynecologic: endometrial, vulvar and cervical stage 1) seen at one of two UNMCCC clinics. Participants were female, English or Spanish speaking, aged 21 or older. To address the need to transition patients into a primary care setting, we stratified participant responses into two groups based on their distance from the center and rurality: (1) Urban & ≤30 miles and (2) Rural or >30 miles. Based on the geographic area of the center, distance was defined as residing in a zip code outside a 30-mile radius or within a 30-mile radius of the center to represent an estimation of the city limits (all urban). Participant zip codes were also used to classify their residence as rural or urban using RUCA codes. [23] These criteria were selected in order to characterize patients eligible for future inclusion in nurse-navigated care coordination intervention. The University of New Mexico Human Research Protections Office approved the protocol for this study.

### 2.2. Survey Instrument

We developed a survey instrument with four domains: (1) health status and guideline concordance (e.g., the degree to which patients were up to date with recommended preventive care services); (2) primary healthcare utilization, including the proportion of patients with an established primary care provider; (3) cancer care access and perceived burden; and (4) receipt of a survivorship care document. Where applicable, the survey instrument aligned with The Centers for Disease Control and Prevention Behavioral Risk Factor Surveillance System (BRFSS) data [24].

### 2.3. Data Collection

Patients scheduled in the outpatient Breast and Gynecologic Oncology clinics were identified using medical record information and pre-screened for eligibility. Selected patients were asked to participate and further assessed for eligibility. Once they consented, eligible participants completed the survey in-person at the time of their visit or by phone at a later prearranged time based on the patient’s preference. Additionally, participants could opt into a weekly drawing for a $50 merchandise card. Between October 2018 and April 2019, we identified 191 scheduled appointments through prescreening, of which 6.8% were a cancellation/no-show, 11% were not approached, and 2.6% were ineligible. Of the 152 eligible women approached, 150 completed the survey for a 98.7% response rate. Data were managed using REDCap (Research Electronic Data Capture) electronic data capture tools hosted at the research institution [25].

### 2.4. Analysis Plan

Descriptive summaries were performed and analyzed to explore the potential relationships between demographic and patient-specific attributes, as well as to characterize the patient population. Respondent demographics included age, race, ethnicity, spoken language, education, household income, cancer type, and rural residence. We also examined healthcare status and primary healthcare utilization by assessing disease comorbidities, smoking status, cancer prevention and screening behaviors, established primary care and utilization in the past five years, and distance to care. Cancer care access and perceived burden for the appointment, coinciding with the survey administration, included patient-reported hardships resulting from loss of work, overnight stays, family/friend assistance, and patient-reported duress. Patient distress from arranging and traveling to the appointment were assessed on a scale from 0–100, which was then collapsed into 4 levels: None (0), Low (1–33), Medium (34–66) and High (67–100). Questions of receipt of survivorship care documents were also included. Categorical data were presented as frequencies (percent), while continuous data were shared as means, standard deviation (SD). To further inform the development of a survivorship care transition model, in addition to presenting the data for the entire sample, we stratified participant responses into two groups based on their distance from UNMCCC, including those considered urban and traveling ≤30 miles, and those who were rural and/or urban residents, traveling >30 miles. Due to the small proportion of urban residents traveling >30 miles, in addition to cancer and healthcare resources, we combined all >30 miles residents regardless of rurality. This survey was not designed for hypothesis testing; therefore, no *p*-values were reported. The data analysis for this paper was generated using SAS software, Version 9.4 of the SAS System for Windows.

## 3. Results

### 3.1. Respondent Demographics

Among the 150 participants who completed the survey, the majority reported a travel distance ≤30 miles and resided in an urban area (N = 98). Data from rural residents (n = 39) and those from urban areas traveling in excess of 30 miles (n = 13) were presented together, as response patterns consistently demonstrated patient barriers stemming from the distance travelled and access to care. Findings where this was not the case are specified in this results section. Self-reported race/ethnicity aligned with statewide and UNMCCC patient estimates (41.6% Hispanic and 15.3% American Indian) consistent with our sampling goal to represent our catchment area. Participants’ cancer types were equally divided between gynecologic (52.7%) and breast (47.3%) cancers. Over one third of participants reported less than USD 25,000 annual household income (36.7%). See Table 1 for demographic characteristics.

### 3.2. Health Status and Guideline Concordance

Shown in Table 2, the vast majority of respondents (78%) reported at least one comorbidity, with almost one third (30.7%) indicating a current diagnosis of hypertension. The burden of comorbidities appeared evenly distributed across travel distance groups. However, respondents who reported current tobacco use as “every day” were higher among participants in the ≤30 miles category (9.2% vs. 1.9%).

With regard to self-reported guideline concordance for recommended primary care services, we found variable patterns among respondent categories. For some common preventive screenings, participants in the >30 miles category reported higher rates. In response to questions about colorectal cancer screening, women traveling a further distance had higher levels of colonoscopy receipt (81.8% vs. 73.5%), as well as completion of a blood stool test (47.7% vs. 33.7%). Similarly, women also reported slightly higher rates of undergoing a mammogram (94.4% vs. 90%).

### 3.3. Primary Health Care Utilization

Overall, a surprisingly high proportion of participants indicated having a current PCP (88%), the vast majority of which were identified as either Family Practice or Internal Medicine (84.7%). Women across both travel distance categories reported regular primary care utilization as over half (57.4%), indicating that they saw a PCP 3 to 4 times per year or more. Participants in the >30 miles category indicated a longer travel time needed to see a PCP with 13.6% indicating a travel time of over 2 h for such a visit compared to only 1.1% for those in the ≤30 miles category.

### 3.4. Cancer Care Access and Perceived Burden

Participants indicated minimal distress with regard to arranging an appointment (58.1% selecting 0 on a 100 point scale) and getting to the UNMCCC (53.0% selecting 0 on a 100 point scale). Responses among participants in the >30 miles category, however, reflect a higher degree of perceived burden in terms of missing work to come to the appointment (31.4% vs. 22.9%), having a family member or friend missing work to accompany them on their visit (28.8% vs. 8.2%), staying overnight for the visit due to travel times or fatigue (43.1% vs. 0) and being driven by a family member or friend (48.1% vs. 31.6%).

### 3.5. Receipt of Survivorship Care Document

Lastly, we included BRFSS questions pertaining to the receipt of a survivorship care document (SCD). Response patterns were similar across travel distance categories and appeared to be consistent with other nationally reported rates of SCD receipt. The vast majority of respondents (88.7%) affirmed receiving instructions from a healthcare professional regarding who they should see for routine cancer check-ups after completing treatment, yet, only 42% reported the receipt of a written SCD.

## 4. Discussion

We conducted this survey to provide context for developing a sustainable statewide Oncology/PCP care coordination model and survivorship program to enhance care for underserved multi-ethnic, multi-cultural, rural and urban cancer patients in New Mexico. The need for such coordinated care seems obvious when considering the striking disparities in cancer incidence and outcomes among the state’s populations. For example, data derived from the New Mexico Tumor Registry demonstrates that while non-Hispanic White women in New Mexico have the highest rates of breast cancer in the state, over the past four decades the incidence of breast cancer has steadily increased in Hispanic and American Indian women, and these women present with higher stages of disease and have poorer overall survival rates [26]. Importantly too, in contrast to non-Hispanic White women, Hispanic and American Indian women have higher rates of colorectal (AI 31.4%, Hispanic 30.6%, NHW 27.8%), kidney (AI 15.1%, Hispanic 13.1%, NHW 8.7%), gastric (AI 7.8%, Hispanic 6.9%, NHW 3.2%), and gall bladder (AI 7.2%, Hispanic 4.4%, NHW 1.9%) cancers.

The survey’s findings presented in this study were therefore surprising, as the vast majority of patients reported having established primary care providers (88%), a finding at odds with the noted cancer disparities. Moreover, previous qualitative findings [27], and current state-level data show that virtually all of New Mexico’s counties (32 out of 33) are designated as either “whole” or “partial” primary care Health Professional Shortage Areas [28]. These conflicting evidentiary patterns are significant, as a number of research studies highlight the ways in which care disparities stemming from provider shortages in rural areas contribute to worse cancer care outcomes for underserved patients [16,29].

To verify such findings, we gathered additional information in a brief QI assessment at the conclusion of patients’ cancer treatments. Those who participated in the QI assessment indicated that the nature of the relationship with their PCP was often more uncertain than indicated in survey responses. In some cases, patients had access to a primary care clinic (e.g., a Federally Qualified Health Center or urgent care clinic) rather than a specific provider. In others, they reference a PCP that has left their clinic, and they have yet to re-establish care with a new provider. Such insights reveal the importance of gathering specific data on the context of care access as precursors to engaging PCPs in continuous and coordinated care management for cancer patients [30].

These findings serve as a reminder that researchers and oncology care providers cannot simply rely on access to community PCPs to address gaps in care along the cancer care continuum. The results emphasize disparities for rural patients that align with broader scientific discussions including elevated rates of health comorbidities, poverty, and treatment- and travel-related distress when compared to urban counterparts [29], as well as those that affect racial/ethnic minority communities. While the majority of results in this article are similar for rural residing patients and others who traveled ≤30 miles for cancer care, these listed disparities indicate that interventions for rural cancer survivors will need to respond to more complex social dynamics affecting remote patients.

### 4.1. Implications

**Figure 1:**
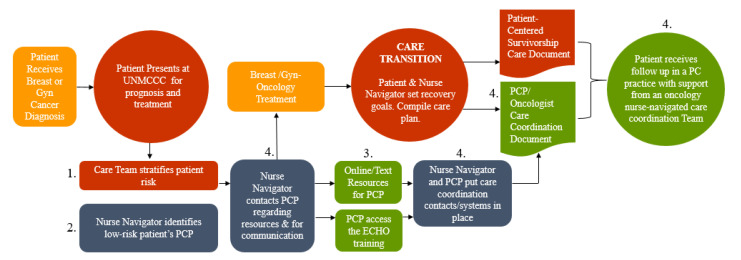
Oncology and Primary Care Provider Care Coordination Model.

These findings represent an important first step toward the development of a sustainable statewide Oncology/PCP care coordination model that is sufficiently flexible to meet the needs of ethnically/racially and geographically diverse cancer patients in unique, localized communities. While there is no conclusive evidence supporting the superiority of one model over another, our review of the scientific literature pinpoints specific features that are critical to the creation of a care coordination model that has the capacity to render timely and effective care for diverse and complex patients in real world primary care settings. These features, addressed numerically on Figure 1, include:

1. Facilitated patient risk stratification [31,32];
2. Navigated Care Coordination including assistance locating a PCP in the event that the patient does not have one [33];
3. Enriched PCP education and knowledge of cancer survivorship [34];
4. Enhanced communication between oncologists and PCPs at the onset of a patient’s cancer treatment, [1] including use of SPDs [35] over the course of their coordinated care [15,27,36].

Navigated care coordination models hold promise for increased patient satisfaction [37] due to the provision of patient-centered care, the coordination of services [38], and emotional support and education [39] across the care continuum [40]. Based on these criteria and our survey data, we are developing a model using UNMCCC nurse navigation as a primary point of contact for patients and PCP collaborators (See Figure 1). Patients and PCPs will receive comprehensive SCDs, and PCPs will have access to online cancer survivorship and surveillance training resources. By creating multimodal care delivery strategies, we will open pathways for bi-directional communication ensuring the relevance, acceptability, and feasibility of care coordination for PCPs who are taking on a higher level of treatment and surveillance for cancer survivors.

### 4.2. Limitations

This survey is limited by the small, convenience sample of an adherent survivor population in New Mexico and may not be generalizable to other localities with different population compositions, cancer incidence patterns, and healthcare workforce levels. However, we achieved a high response rate, with participants accurately reflecting the multi-ethnic, geographical diversity of patients served by the UNMCCC. We likewise recognize that cancer survivorship and oncology/PCP care coordination are complex, multifactorial issues, and suggest that additional research is warranted to ensure that any care coordination model implemented is truly effective, patient-centered, and mutually beneficial for PCP care collaborators.

## 5. Conclusions

When considering patterns of cancer disparities in New Mexico, and the potential of oncology-/PCP-coordinated cancer care to improve patient care, this research demonstrates two important findings. First, disparities stemming from race/ethnicity, cultural diversity, distance, financial hardship, and inadequate psychosocial and specialized services for rural cancer patients are pervasive in New Mexico. These disparities must be addressed in order to provide equitable care for ethnically, racially, and culturally diverse cancer patients in both rural and urban areas. Second, while sometimes under-resourced or understaffed, and lacking in cancer survivorship specific training, PCPs are key providers for oncology patient care. Oncologists have an opportunity to respond to contextual factors that perpetuate cancer disparities by partnering meaningfully with PCPs to improve follow-up care for diverse low-risk cancer patients.

## Figures and Tables

**Table 1:** Respondent Demographics, n (%).

	Total (*n* = 150)	≤30 miles (*n* = 98)	>30 miles (*n* = 52)
Age, Mean, SD	59.2, 11.3	59.0, 11.4	59.7, 11.1
Race			
White	102 (68.0)	73 (74.5)	29 (55.8)
American Indian	23 (15.3)	4 (4.1)	19 (36.5)
Other/Mixed	25 (16.7)	21 (21.4)	4 (7.7)
Hispanic	62 (41.6)	46 (46.9)	16 (31.4)
Language			
English	136 (91.3)	90 (92.8)	46 (88.5)
Spanish	6 (4.0)	5 (5.2)	1 (1.9)
Another language	7 (4.7)	2 (2.1)	5 (9.6)
Out of State	10 (6.7)	0 (0)	10 (19.2)
Education			
Less than high school	12 (8.0)	9 (9.1)	3 (5.7)
High school	40 (26.7)	28 (28.6)	12 (23.1)
Some college	27 (18.0)	16 (16.3)	11 (21.2)
2-year college degree	25 (16.7)	14 (14.3)	11 (21.2)
4-year college degree	22 (14.7)	17 (17.3)	5 (9.6)
Postgraduate degree	24 (16.0)	14 (14.3)	10 (19.2)
HH Income			
Less than USD 9999	16 (10.7)	11 (11.2)	5 (9.6)
USD 10,000–USD 24,999	39 (26.0)	29 (29.6)	10 (19.2)
USD 25,000–USD 49,999	38 (25.3)	20 (20.4)	18 (34.6)
USD 50,000–USD 74,999	22 (14.7)	14 (14.3)	8 (15.4)
More than USD 75,000	29 (19.3)	22 (22.4)	7 (13.5)
Prefer not to answer	6 (4.0)	2 (2.0)	4 (7.7)
Cancer Type			
Endometrial	59 (39.3)	25 (25.5)	34 (65.4)
Cervical	19 (12.7)	13 (13.3)	6 (11.5)
Breast	71 (47.3)	59 (60.2)	12 (23.1)
Vulvar	1 (0.7)	1 (1.0)	0 (0)
Rural Residence	39 (26.0)	0 (0)	39 (75.0)

**Table 2:** Healthcare Access and Utilization by Place of Residence and Travel Distance to Cancer Center, *n* (%).

Construct		Total (*n* = 150)	≤30 miles (*n* = 98)	>30 miles (*n* = 52)
2 (2.0)	Number of comorbidities			
	0	33 (22.0)	23 (23.5)	10 (19.2)
	1	50 (33.3)	32 (32.7)	18 (34.6)
	2	32 (21.3)	21 (21.4)	11 (21.2)
	3+	35 (23.3)	22 (22.4)	13 (25.0)
	Prediabetes	19 (12.7)	11 (11.2)	8 (15.4)
	Diabetes	32 (21.3)	19 (19.4)	13 (25.0)
	Hypertension	46 (30.7)	26 (26.5)	20 (38.5)
	Stroke	4 (2.7)	2 (2.0)	2 (3.8)
	Angina or coronary heart disease	4 (2.7)	4 (4.1)	0 (0)
	Heart attack	4 (2.7)	3 (3.1)	1 (1.9)
	Asthma	21 (14.0)	12 (12.2)	9 (17.3)
	Arthritis, Rheumatoid Arthritis, Gout, Lupus, or Fibromyalgia	38 (25.3)	23 (23.5)	15 (28.8)
	Depressive disorders	33 (22.0)	24 (24.5)	9 (17.3)
	Kidney disease	4 (2.7)	4 (4.1)	0 (0)
	Chronic Obstructive Pulmonary Disease, emphysema, or chronic bronchitis	4 (2.7)	4 (4.1)	0 (0)
	Any other types of cancer	10 (6.7)	4 (4.1)	6 (11.5)
	Other disease	26 (17.3)	19 (19.4)	7 (13.5)
	Current smoker (cigarettes)			
	Every day	10 (6.7)	9 (9.2)	1 (1.9)
	Some days	2 (1.3)		0 (0)
	Not at all	138 (92.0)	87 (88.8)	51 (98.1)
	Ever had a colonoscopy	97 (76.4)	61 (73.5)	36 (81.8)
	Ever had a blood stool test using a home kit	49 (38.6)	28 (33.7)	21 (47.7)
	Ever had a mammogram	61 (92.4)	27 (90.0)	34 (94.4)
Primary Health Care Utilization:	Have PCP	132 (88.0)	89 (90.8)	43 (82.7)
	PCP Type			
	Family practice/internal medicine doctor	111 (84.7)	76 (85.4)	35 (83.3)
	OB-GYN	3 (2.3)	2 (2.2)	1 (2.4)
	Nurse Practitioner/Physician’s Assistant	16 (12.2)	11 (12.4)	5 (11.9)
	Other	1 (0.8)	0 (0)	1 (2.4)
	PCP visits in past 5 years			
	Monthly	13 (8.7)	8 (8.2)	5 (9.6)
	3–4 times per year	73 (48.7)	45 (45.9)	28 (53.8)
	Once per year	51 (34.0)	37 (37.8)	14 (26.9)
	Every few years	13 (8.7)	8 (8.2)	5 (9.6)
	Travel time to PCP			
	Less than 30 min	107 (79.9)	77 (85.6)	30 (68.2)
	30 min to 1 h	16 (11.9)	9 (10.0)	7 (15.9)
	1 h to 1.5 h	3 (2.2)	2 (2.2)	1 (2.3)
	1.5 to 2 h	1 (0.7)	1 (1.1)	0 (0)
	2 to 2.5 h	2 (1.5)	0 (0)	2 (4.5)
	More than 2.5 h	5 (3.7)	1 (1.1)	4 (9.1)
Cancer Care Access and Perceived Burden (Today’s Appointment):	Distress: Arranging Appointment (Scale: 1–100)			
	None (0)	86 (58.1)	56 (58.3)	30 (57.7)
	Low (1–33)	53 (35.8)	33 (34.4)	20 (38.5)
	Medium (34–66)	5 (3.4)	3 (3.1)	2 (3.8)
	High (67–100)	4 (2.7)	4 (4.2)	0 (0)
	Distress: Travel to Appointment (Scale: 1–100)			
	None (0)	79 (53.0)	50 (51.0)	29 (56.9)
	Low (1–33)	53 (35.6)	38 (38.8)	15 (29.4)
	Medium (34–66)	13 (8.7)	8 (8.2)	5 (9.8)
	High (67–100)	4 (2.7)	2 (2.0)	2 (3.9)
	Missed work to come to appointment	38 (25.9)	22 (22.9)	16 (31.4)
	Family member or friend missed work	23 (15.4)	8 (8.2)	15 (28.8)
	Overnight stay for appointment	22 (14.8)	0 (0)	22 (43.1)
	Transportation to appointment			
	Drove self	86 (57.3)	62 (63.3)	24 (46.2)
	Driven by a family member/friend	56 (37.3)	31 (31.6)	25 (48.1)
	Ride service	6 (4.0)	3 (3.1)	3 (5.8)
	Public transportation	2 (1.3)	2 (2.0)	0 (0)
Receipt of Survivorship Care Document:	Ever received instructions about where to return or who to see for routine cancer check-ups after completing treatment for cancer			
	No	15 (10.0)	10 (10.2)	5 (9.6)
	Yes	133 (88.7)	87 (88.8)	46 (88.5)
	Do not know/Not sure	2 (1.3)	1 (1.0)	1 (1.9)
	Ever given a written summary of all the cancer treatments received			
	No	69 (46.0)	44 (44.9)	25 (48.1)
	Yes	63 (42.0)	42 (42.9)	21 (40.4)
	Don not know/Not sure	18 (12.0)	12 (12.2)	6 (11.5)
